# Polyethyleneimine-interlayered silica-core quantum dot-shell nanocomposites for sensitive detection of *Salmonella typhimurium via* a lateral flow immunoassay†

**DOI:** 10.1039/c9ra09252h

**Published:** 2020-01-14

**Authors:** Bo Zhang, Xingsheng Yang, Xiaoxian Liu, Juan Li, Chongwen Wang, Shengqi Wang

**Affiliations:** School of Public Health, Jilin University Changchun 130021 PR China li_juan@jlu.edu.cn; College of Life Sciences, Anhui Agricultural University Hefei 230036 PR China wangchongwen1987@126.com sqwang@bmi.ac.cn; Beijing Institute of Radiation Medicine Beijing 100850 PR China; Department of Pharmacy, Peking Union Medical College Hospital, Chinese Academy Medical Sciences & Peking Union Medical College Beijing 100730 PR China

## Abstract

Herein, we synthesized high-performance SiO_2_–core quantum dot (QD)–shell nanocomposites (SiO_2_@PEI-QDs) using the polyethyleneimine (PEI)-mediated adsorption method. Cationic PEI was used to form a positively charged interlayer on the SiO_2_ core, which achieved a dense adsorption of carboxylated QDs to form a shell of QDs and maintained a good dispersibility of the nanocomposite. The SiO_2_@PEI-QDs showed excellent stability and high luminescence, and served as high-performance fluorescent labels for the detection of bacteria when used with the lateral flow immunoassay (LFA) technique. An SiO_2_@PEI-QD-based LFA strip was successfully applied to rapidly detect *Salmonella typhimurium* in milk samples with a low limit of 5 × 10^2^ cells per mL.

## Abstract 2

A novel type of SiO_2_-core QDs-shell nanomaterial was fabricated and utilized to prepare bright fluorescent nanotags for fluorescent lateral flow strip.

## Graphical abstract

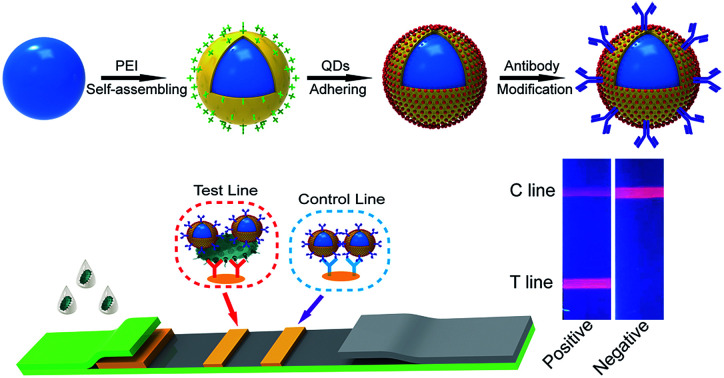

*Salmonella typhimurium* (*S. typhi*) is one of main foodborne pathogens affecting humans and animals worldwide; it causes great damage to public health, high mortality, and high economic loss.^1,2^ Current mature methods for detecting *S. typhi* primarily include the conventional plate culturing, polymerase chain reaction, DNA sequencing, and enzyme-linked immunosorbent assay techniques.^2–5^ However, these methods have several inherent shortcomings, such as requiring tedious procedures and long testing times, multistep sample pretreatment, expensive equipment, and skilled personnel, for the rapid detection of bacteria.^6^ Thus, a sensitive and convenient technique for rapid detection of pathogenic bacteria must be developed.

The lateral flow immunoassay (LFA) technique has become one of the most efficient point-of-care testing tools because of its distinct advantages of simple operation, rapid analysis, low cost, and flexibility for different tested substances.^7–9^ Quantum dots (QDs) as novel fluorescent labels are used in LFAs to improve detection sensitivity and quantitative ability owing to their superior optical properties including photostability, strong luminescence, and narrow fluorescence emission spectral peaks.^10–12^ However, QDs are also subject to several key problems during bioanalysis, such as their tendency to easily aggregate, instability in complex solutions, and lack of biocompatibility.^13^ These problems can be overcome by combining QDs and other support materials, such as latex beads,^14^ SiO_2_,^15^ polymeric microbeads,^16^ and Fe_3_O_4_,^17^ into one micro- or nanosphere. However, most of the reported QD composites have dimensions generally greater than 300 nm and the methods used to synthesize them are complex, which have greatly restricted their application in LFA systems for the detection of bacteria.

Here, we report a facile polyethyleneimine (PEI)-mediated adsorption strategy to fabricate PEI-interlayered SiO_2_–core QD–shell nanomaterials (SiO_2_@PEI-QDs) displaying controllable dimensions, monodispersity, and excellent fluorescence. PEI was employed to form an electropositive thin interlayer on the surface of SiO_2_ NPs not only to achieve a dense adsorption of CdSe/ZnS-MPA QDs as QD shells but also to maintain the stability of the nanostructure in the solution. Our results revealed the performance and stability of SiO_2_@PEI-QDs and demonstrated that the proposed nanocomposites can act as advanced fluorescent nanotags for QD-based LFA strips. The sensitivity of an SiO_2_@PEI-QD-based LFA strip for the detection of *S. typhi* was measured to be as low as 5 × 10^2^ cells per mL. To the best of our knowledge, this work was the first to use high-performance SiO_2_-QD nanocomposites as fluorescent labels for LFA-based detection of bacteria.

The fabricated SiO_2_@PEI-QDs were composed of three parts: SiO_2_ NPs with dimensions of 150 nm as a hydrophilic support core to provide good dispersity, a thin layer of PEI as an electropositive linker, and dense carboxylated QDs forming a shell of QDs to generate high luminescence and surface sites for antibody conjugation. Herein, we chose SiO_2_ NPs with dimensions of 150 nm as the core material because of their uniform size and homogeneous nanostructures, easy preparation, and high stability in complex solutions. CdSe/ZnS-MPA QDs were chosen to form the outer shell of QDs because of the excellent fluorescence properties and numerous surface carboxyl groups of the MPA-modified shell for subsequent antibody coupling.^18^

High-performance SiO_2_@PEI-QDs with a typical core–shell nanostructure were fabricated in three steps, as shown in Scheme 1a. First, monodisperse SiO_2_ NPs were synthesized according to a modified Stöber method as the stable core. Second, PEI-coated SiO_2_ NPs (SiO_2_@PEI) were prepared by dispersing 10 mg of SiO_2_ NPs in an aqueous PEI solution (0.5%, v/v) under sonication for 30 min, during which the cationic polymer PEI quickly self-assembled on the negatively charged SiO_2_ surface.^19–21^ SiO_2_@PEI NPs were collected by carrying out centrifugation, and then dispersed in 5 mL of deionized water. Finally, 1 mL of the as-prepared SiO_2_@PEI NPs was added to 100 mL of a carboxyl-functionalized CdSe/ZnS QD solution (0.1 nM) and sonicated for 1 h to form SiO_2_@PEI-QDs *via* electrostatic interaction. The resulting SiO_2_@PEI-QDs were collected by carrying out centrifugation, and then stored in 10 mL of deionized water for further use.

**Scheme 1:**
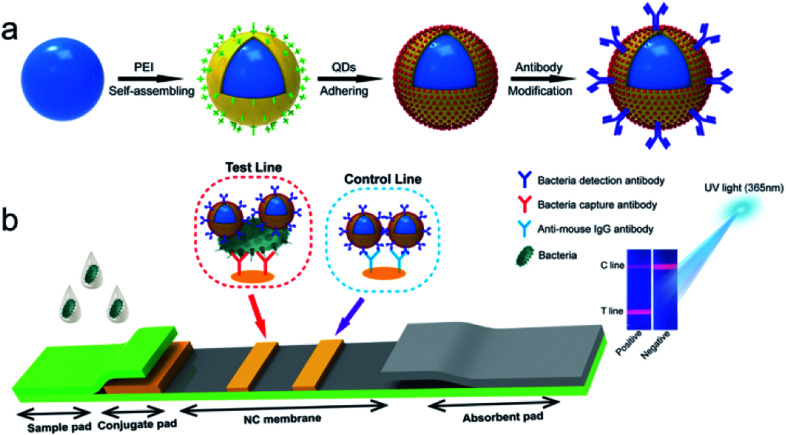
(a) Schematic of the synthesis of antibody-modified SiO_2_@PEI-QDs. (b) Schematic of the quantitative detection of *S. typhi* using an SiO_2_@PEI-QD-based fluorescent LFA strip.

Transmission electron microscopy (TEM) analysis was performed to verify the morphology of the as-synthesized nanocomposites. As shown in Fig. S1a,† the as-prepared SiO_2_ NPs displayed uniforms dimensions of 150 nm and good dispersibility. The TEM image acquired of commercial CdSe/ZnS-MPA QDs is shown in Fig. S1b.† The average particle dimension of the CdSe/ZnS-MPA QDs was approximately 12 nm. After coating SiO_2_ with PEI by carrying out sonication, the resultant SiO_2_@PEI NPs still exhibited good dispersity (Fig. 1a). The acquired high-resolution TEM (HRTEM) image of one of these NPs clearly showed that the thickness of the PEI layer was about 8 nm (Fig. 1c). Fig. 1b and d show the acquired low- and high-magnification TEM images of the fabricated SiO_2_@PEI-QDs. Dense CdSe/ZnS QDs were decorated uniformly on the surfaces of the SiO_2_@PEI NPs. Moreover, the average diameter of the nanocomposites increased from 150 nm to 182 nm after the QD–shell formation. The energy dispersive spectroscopy (EDS) mapping technique was used to confirm the elemental composition of the QD-coated SiO_2_ nanocomposites. As shown in Fig. 1e, high densities of Cd (green), Se (purple), Zn (red), and S (yellow) were found to surround the Si core (blue). This finding indicated the typical core–shell nanostructure of SiO_2_@PEI-QDs. All of the TEM and EDS results confirmed the successful preparation of the SiO_2_@PEI-QDs. Note that the branched PEI effectively attached to the surfaces of the SiO_2_ NPs in the aqueous solution and easily realized the full surface amino modification of SiO_2_ NPs, which was the key to achieving a dense adsorption of CdSe/ZnS-MPA QDs as uniform shells of QDs. Though other cationic polymers such as poly(diallyl dimethylammonium chloride) can also self-assemble on the surfaces of SiO_2_ NPs to adsorb QDs, the pH value of the reaction solution in these cases needed to be precisely controlled.^15^

**Fig. 1:**
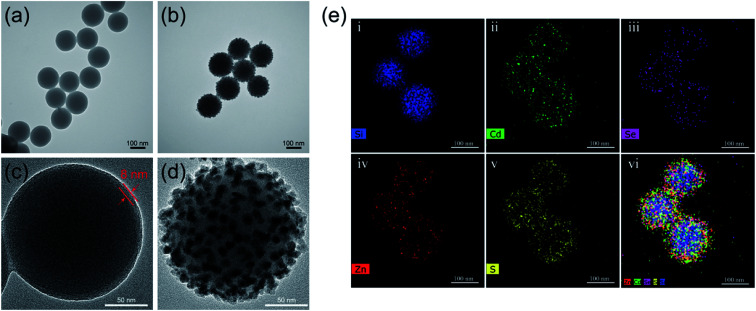
Characterizations of the morphologies and elemental compositions of the synthesized SiO_2_@PEI-QDs. (a and b) TEM images of (a) SiO_2_@PEI NPs and (b) SiO_2_@PEI-QDs. (c and d) Magnified TEM images of (c) a single SiO_2_@PEI NP and (d) a single SiO_2_@PEI-QDs NP. (e) Elemental mapping images of SiO_2_@PEI-QDs.

As shown in Fig. 2a and b, the zeta potential of the SiO_2_@PEI-QDs clearly changed during the course of their synthesis. The zeta potential dramatically increased from −44.7 mV for the SiO_2_ NPs to +52.5 mV for SiO_2_@PEI, *i.e.*, after the coating of the positively charged PEI layer; it then decreased to +27.8 mV for the SiO_2_@PEI-QDs, due to the numerous adsorbed negatively charged QDs. These results confirmed that the formation of the SiO_2_@PEI-QDs was based on PEI-mediated electrostatic adsorption. We next studied the fluorescence properties of the SiO_2_@PEI-QDs. Fig. 2c(i) and (ii) show SiO_2_, SiO_2_@PEI, and SiO_2_@PEI-QD suspensions under visible and 365 nm-wavelength ultraviolet (UV) light, respectively. A bright-red optical emission was observed for SiO_2_@PEI-QDs excited with a UV light source, whereas SiO_2_ and SiO_2_@PEI groups showed no fluorescence signal. Fig. 2d shows the corresponding fluorescence emission spectra of the as-synthesized nanocomposites; these spectra revealed the superior fluorescence performance of the SiO_2_@PEI-QDs. Moreover, the fluorescence intensity of SiO_2_@PEI-QDs remained unchanged for three months when stored in ethanol (Fig. S2†), demonstrating their high fluorescence stability. The outstanding fluorescence properties and stability enabled SiO_2_@PEI-QDs to, as described next, act as high-performance fluorescent labels for LFA strip-based detection of bacteria.

**Fig. 2:**
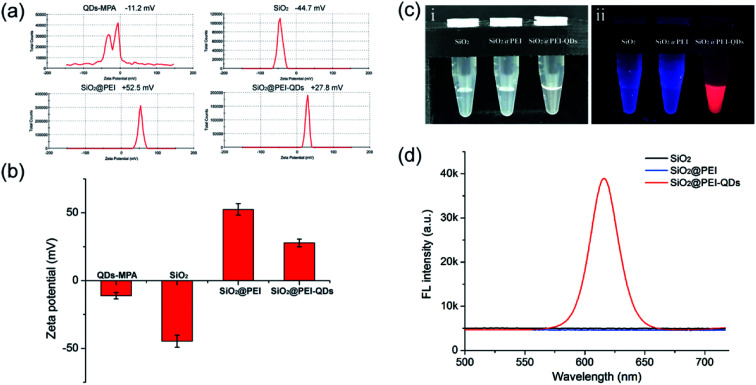
Characterizations of the electric charge and fluorescence properties of the synthesized SiO_2_@PEI-QDs. (a) Zeta potentials and (b) statistical analysis of the zeta potentials of the as-obtained nanocomposites at different stages of their synthesis. (c) Photographs of SiO_2_, SiO_2_@PEI, and SiO_2_@PEI-QDs suspensions under visible (i) and UV light (ii). (d) Fluorescence emission spectra of these particles in deionized water.

Scheme 1b illustrates the experimental principle of the use of SiO_2_@PEI-QD-based fluorescence LFA to detect bacteria. The strip system consisted of four independent parts: a sample pad, conjugate pad, nitrocellulose (NC) membrane, and absorbent pad. Anti-*S. typhi* monoclonal antibody and goat anti-mouse IgG antibody were simultaneously dispensed into the NC membrane to form test and control lines, respectively. When the strip was inserted into the sample solution, the solution moved toward the absorbent pad through capillary force. In the presence of *S. typhi*, SiO_2_@PEI-QDs modified with the *S. typhi* monoclonal antibody recognized and bound to the target bacteria and were finally captured on the test zone by forming SiO_2_@PEI-QD–*S. typhi*–antibody sandwich immune complexes. Superfluous immune SiO_2_@PEI-QDs continued to move forward and were immobilized on the control line of the strip. Finally, bacteria were quantitatively analyzed by recording the fluorescence intensity of the test line with a fluorescence reader (with 365 nm-wavelength excitation).

Immuno-SiO_2_@PEI-QDs were prepared by labeling the surface carboxyl groups of SiO_2_@PEI-QDs directly with *S. typhi* antibodies *via* carbodiimine chemistry. The zeta potential value decreased from +27.8 mV for the SiO_2_@PEI-QDs to +12.7 mV for the immuno-SiO_2_@PEI-QDs, suggesting the successful attachment of antibody molecules onto the surfaces of the SiO_2_@PEI-QDs (Fig. S3†). A TEM image of the immuno-SiO_2_@PEI-QDs showed that they remained monodispersed after the antibody modification (Fig. 3a). As shown in Fig. 3b, the antibody-conjugated SiO_2_@PEI-QDs were directly observed using TEM to effectively bind an *S. typhi*. target.

**Fig. 3:**
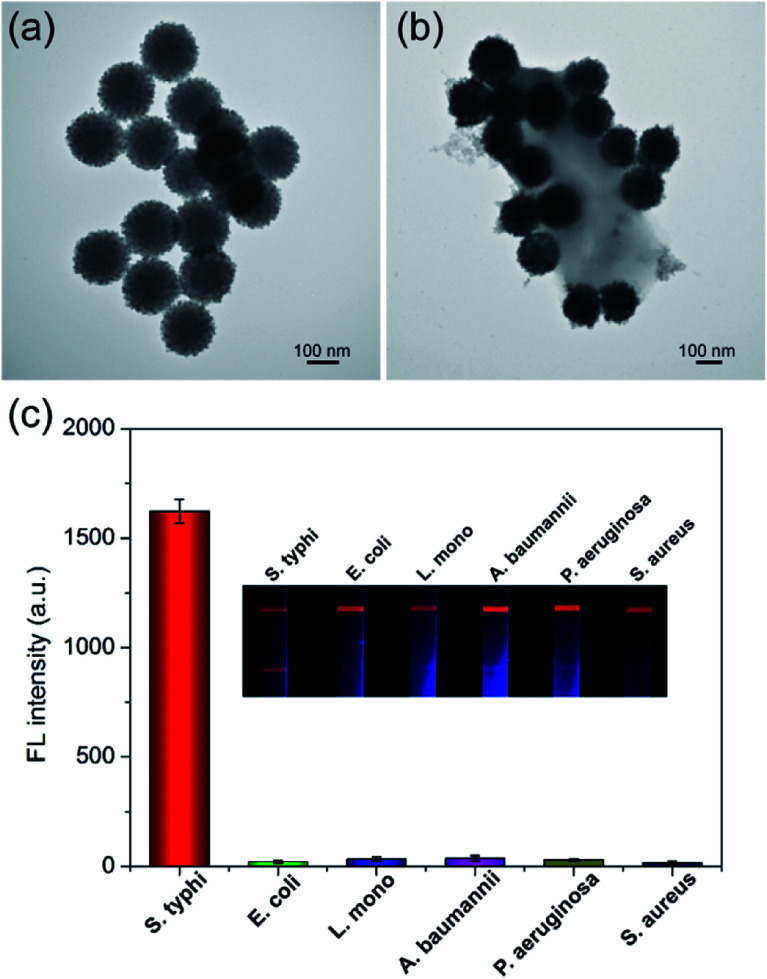
(a and b) TEM images of (a) immuno-SiO_2_@PEI-QDs and (b) an immunocomplex of SiO_2_@PEI-QDs and *S. typhi*. (c) Specificity of the SiO_2_@PEI-QD-based fluorescent LFA strip. The inset shows photographs of the test strips in the presence of *S. typhi* and five interfering bacteria each at a concentration of 10^5^ cells per mL. The error bars represent the standard deviations from three repeats of the experiment.

After preparation of immuno-SiO_2_@PEI-QDs, the important conditions of the LFA detection system were optimized. The running buffer of the LFA strip was first evaluated because of its direct effect on the flow rate of SiO_2_@PEI-QD nanotags and the immune binding efficiency of the test line.^22,23^ As shown in Fig. S4a and b,† the PBST buffer (10 mM, pH 7.4, 1% Tween 20) ensured a smooth delivery of SiO_2_@PEI-QD-bacteria complexes along the strip and achieved the highest signal-to-noise ratio (SNR) on the test line. We then optimized the concentration of the coated antibodies on the test zone. The highest SNR was achieved when the concentration of the *S. typhi* antibody was 0.6 mg mL^−1^ (Fig. S4c†). The selectivity and specificity of the LFA system greatly depended on the specific detection antibody applied to the SiO_2_@PEI-QDs. The performance of the *S. typhi* monoclonal antibody was then tested. A total of 10^5^ cells per mL of *S. typhi* and five other common pathogenic bacteria including *Escherichia coli* (*E. coli*), *Listeria monocytogenes* (*L. mono*), *Acinetobacter baumannii* (*A. baumannii*), *Pseudomonas aeruginosa* (*P*. *aeruginosa*), and *Staphylococcus aureus* (*S. aureus*) were used to test the specificity of the proposed assay. As shown in the inset of Fig. 3c, only *S. typhi* exhibited an evident fluorescence signal on the test line, whereas all nontarget bacteria groups showed no fluorescence signal on the same area. Moreover, bright fluorescence control lines appeared on all of the strips, indicating that the LFA strips were working correctly. The corresponding fluorescence intensities of the test lines were recorded. The results indicated the good selectivity of the SiO_2_@PEI-QD-based strip for the detection of *S. typhi* (Fig. 3c). Moreover, the stability and anti-interference ability of the SiO_2_@PEI-QD-based strip was evaluated by setting out to detect *S. typhi* in untreated milk and tap water samples. As shown in Fig. S5,† the milk and tap water groups as well as PBS group generated strong fluorescence signals, indicating that the proposed strip can work well in real food samples.

We evaluated the detection sensitivity of the proposed assay in milk samples under the optimized conditions. Fig. 4a(i) and (ii) show photographs and fluorescence pictures of the SiO_2_@PEI-QD-based strip used to test milk samples spiked with various concentrations of *S. typhi* (10^7^ cells per mL to 0 cells per mL). Under 365 nm-wavelength UV excitation, the red fluorescence band of the test line became darker with decreasing concentration of *S. typhi* in the milk samples. No evident fluorescence signal was observed for the blank group. The visualization limit of the fluorescence signal of the SiO_2_@PEI-QD-based strip for *S. typhi* was 10^3^ cells per mL. The corresponding test line fluorescence intensities were recorded using a fluorescent strip reader. The fluorescence results were analyzed by plotting the corresponding fluorescence intensities of the test lines as a function of *S. typhi* concentration to produce a calibration curve (Fig. 4b). A linear relationship was observed here within the range 1 × 10^4^ to 5 × 10^2^ cells per mL for *S. typhi* (inset of Fig. 4b). The limit of detection (LOD) of the SiO_2_@PEI-QD-based strip was estimated to be 5 × 10^2^ cells per mL, with the LOD defined as the 3 : 1 threshold ratio with respect to the blank signal.^24–26^ These results revealed that the proposed LFA strip with SiO_2_@PEI-QDs as the fluorescence label displayed high sensitivity and a wide dynamic range for *S. typhi* detection. For comparison, the *S. typhi* concentrations in test milk samples were also confirmed using the traditional plate counting method. As displayed in Fig. S6,† the number of bacterial colonies grown on the plates was consistent with the LFA-strip detection results, indicating the good accuracy of the strip based on SiO_2_@PEI-QDs. Moreover, the proposed SiO_2_@PEI-QD-based strip enabled a rapid detection of *S. typhi*, specifically within 15 minutes, whereas the plate counting method generally requires 8–24 h.^27,28^ The reproducibility of the SiO_2_@PEI-QD-based LFA strip was also investigated by testing milk samples containing various concentrations of *S. typhi*. Five independent tests were conducted to measure *S. typhi* samples at concentrations of 10^5^ and 10^3^ cells per mL. As shown in Fig. S7,† the proposed assay exhibited high repeatability and reliability.

**Fig. 4:**
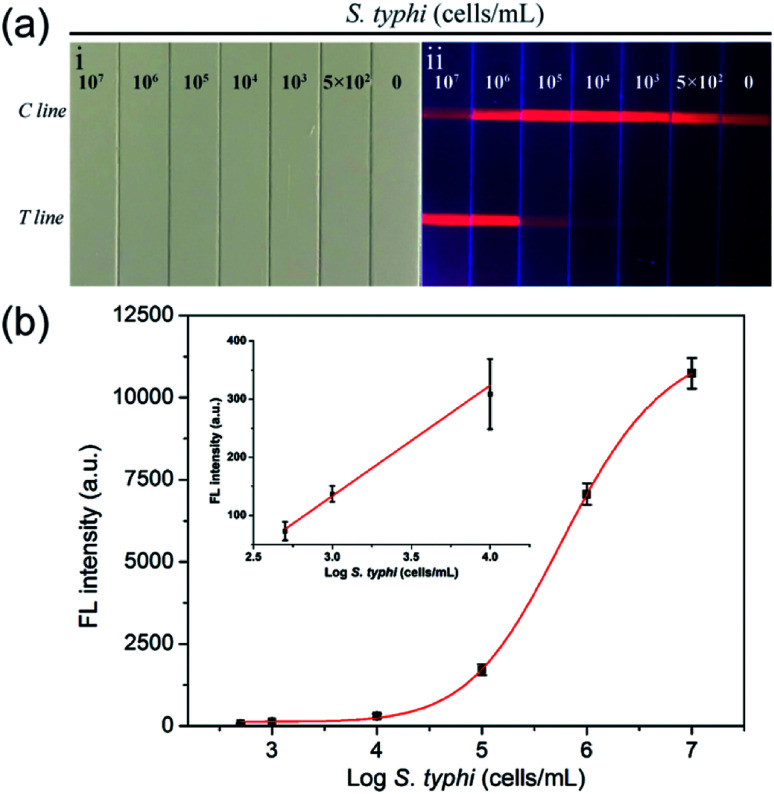
(a) Photographs (i) and fluorescence pictures (ii) of the SiO_2_@PEI-QD-based fluorescent LFA strip used for detecting *S. typhi*. (b) Corresponding test line intensities and calibration curve as a sigmoidal function of the concentration of *S. typhi* within the range 10^7^ to 0 cells per mL. The inset shows the linear relationship of the data in the low-concentration range.

To directly compare the detectability of the proposed SiO_2_@PEI-QDs strip with that of a common fluorescent LFA strip, we prepared a QD nanocomposite-based LFA strip by using the same *S. typhi* detection antibody but replaced SiO_2_@PEI-QDs with commercial QD nanocomposites. As shown in Fig. S8a,† a TEM image acquired of the commercial QD nanocomposites showed them also containing dozens of QDs per particle, but with dispersibility and uniformity levels inferior to those of SiO_2_@PEI-QDs. As shown in Fig. S8b,† the red fluorescence test lines of the QD nanocomposite-based strip for *S. typhi* were observed with the naked eye at a concentration of 1 × 10^4^ cells per mL. The corresponding calibration curve was constructed, as shown in Fig. S8c.† By comparison, the LOD of the SiO_2_@PEI-QDs strip was 20 times lower than that of the commercial QD nanocomposite-based LFA strip for *S. typhi* detection. The excellent performance of the assay for the detection of bacteria can be attributed to the advanced SiO_2_@PEI-QDs used in the LFA system. These nanocomposites showed numerous shells of QDs, monodispersity, good stability, and a highly reproducible structure. In contrast to other recently reported LFA methods used for detecting bacteria, the proposed SiO_2_@PEI-QDs strip showed better sensitivity (Table 1). Moreover, the SiO_2_@PEI-QD-based LFA strip can be easily applied for rapid detection of other pathogenic microorganisms by using specific monoclonal antibodies.

**Table 1:** Overall performance of the SiO_2_@PEI-QD-based fluorescent LFA strip compared with other respiratory virus detection techniques

Detection method	Bacteria	Detection limit (cells per mL)	Sample	Reference
Colorimetric LFA	*Salmonella*	10^3^	Milk	Hwang *et al.* 2016 (ref. 29)
Colorimetric LFA	*B. cereus*	10^4^	PBS	Kong *et al.* 2017 (ref. 30)
Up-converting phosphor LFA	*S. typhi*	10^4^	Various foods	Zhao *et al.* 2017 (ref. 31)
Colorimetric LFA	*E. coli O157*	4.5 × 10^3^	Milk	Zhu *et al.* 2018 (ref. 32)
Fluorescent LFA	*E. coli O157*	3 × 10^3^	Beef, milk	Li *et al.* 2019 (ref. 33)
Fluorescent-magnetic LFA	*S. typhi*	3.75 × 10^3^	Milk, blood	Hu *et al.* 2019 (ref. 34)
Fluorescent LFA	*S. typhi*	5 × 10^2^	Milk	This work

In summary, a novel type of SiO_2_–core QD–shell nanomaterial was fabricated and utilized to prepare bright fluorescent nanotags for LFA strips. By using PEI as the interlayer, numerous carboxyl-functionalized CdSe/ZnS QDs were rapidly and firmly self-assembled on the surfaces of SiO_2_ NPs, forming a stable nanocomposite with good dispersity, a highly reproducible structure, and high luminescence. Based on the quantitative analysis of *S. typhi* with a detection limit of as low as 5 × 10^2^ cells per mL, our results further demonstrated that these SiO_2_@PEI-QDs can be used as high-performance fluorescent labels for LFA-based detection of bacteria. We believe that the proposed SiO_2_@PEI-QD-based LFA strip has great potential for rapid and sensitive detection of pathogens in real samples.

## Conflicts of interest

The authors declare no conflict of interest.

## Supplementary Material

RA-010-C9RA09252H-s001
