# Supplementary material for: Polyethyleneimine-interlayered silica-core quantum dot-shell nanocomposites for sensitive detection of Salmonella typhimurium via a lateral flow immunoassay
Source: RSC Adv. 2020 Jan 14;10(5):2483–9. doi: 10.1039/c9ra09252h (PMC9048750; doi:10.1039/c9ra09252h)
Supplement: RA-010-C9RA09252H-s001 [file RA-010-C9RA09252H-s001.pdf]

Electronic Supplementary Material (ESI) for RSC Advances.
This journal is © The Royal Society of Chemistry 2020

Supporting Information

Polyethyleneimine-interlayered silica-core quantum dot-shell nanocomposites for sensitive detection of *Salmonella typhimurium* via lateral flow immunoassay

Bo Zhang[a,d], Xingsheng Yang[b,c], Xiaoxian Liu[b,c], Juan Li[a,*], Chongwen Wang[b,c,*], Shengqi Wang[b,c,*]

[a] School of Public Health, Jilin University, Changchun 130021, PR China.

[b] College of Life Sciences, Anhui Agricultural University, Hefei 230036, PR China.

[c] Beijing Institute of Radiation Medicine, Beijing 100850, PR China.

[d] Department of Pharmacy, Peking Union Medical College Hospital, Chinese Academy Medical Sciences & Peking Union Medical College, Beijing 100730, PR China.

S.1 Experimental section

S1.1 Materials and chemicals

N-(3-dimethyaminopropy)-N′-ethylcarbodiimide hydrochloride (EDC), N-hydroxysulfosuccinimide sodium salt (sulfo-NHS), bovine serum albumin (BSA), 2-(N-morpholino)ethanesulfonic (MES), fetal bovine serum (FBS), tetraethoxysylane (TEOS), and polyethyleneimine branched (PEI, MW 25 kDa) were purchased from Sigma-Aldrich (USA). Mouse monoclonal antibody to *S. typhi* (catalog no. ab8274) was purchased from Abcam (Cambridge, U.K.). Goat anti-mouse IgG was purchased from Sangon Biotech Co., Ltd. (Shanghai, China). Nitrocellulose membrane (UniSart CN95) with 15 μm pore size was obtained from Sartorius (Spain). The sample loading pad, conjugate pad, absorbent pad, and plastic backing card were purchased from Jieyi Biotechnology Co., Ltd. (Shanghai, China).

S1.2 Instruments

The TEM images and HR-TEM images of SiO_2@PEI-QDs were recorded on a Hitachi H-7650 microscope at an accelerating voltage of 80 kV and a Philips Tecnai G2 F20 microscope at an accelerating voltage of 200 kV, respectively. Elemental mapping images were recorded by energy-dispersive X-ray spectroscopy (EDS) using a Philips Tecnai G2 F20 microscope equipped with a STEM unit. The zeta potentials of the synthesized nanocomposites were characterized using Nano-ZS90 Zeta Sizer. Emission spectra of QD-based fluorescent LFA strip were recorded by using a FIC-S1 fluorescent strip reader (Suzhou Hemai, China).

S1.3 Bacteria sample preparation

The standard strain of *S. typhi*, *E. coli*, *S. aureus*, *P. aeruginosa*, *L. mono*, and *A. baumannii* were provided by Beijing Institute of Radiation Medicine. Conventional plate counting method was used to determine the concentrations of bacteria. In brief, bacterial strain was cultivated in 5% sheep blood agar plates at 37 °C in an atmosphere containing 5% CO_2. After 12 h culture, dozens of colonies were picked from the plate, and transferred into 1 mL of PBS solution (10 mM, pH 7.4) as initial bacterial solution. Then, 0.2 mL of bacterial solution was diluted in sterile water for 1×10^5 times, and coated on the blood agar plate at 37 °C. After overnight culture, the number of colony-forming units (CFUs) on the plate was counted. According to the result of CFU counting, the initial bacterial solution was adjusted to concentrations of 10^7 cells/mL for follow-up test.

S1.4 Preparation of SiO_2@PEI-QDs

The monodispersed SiO_2@PEI-QDs with a core-shell nanostructure were fabricated via a simple three-step reaction. First, 150 nm SiO_2 NPs were prepared according to a modified method.[1] In brief, 4 mL of ammonia solution (28%) and 6 mL of deionized water were added to 100 mL ethanol under magnetic stirring (600 rpm/min). Then, 3.7 mL of TEOS was added into the above mixture, and the reaction was kept at room temperature for 4h. Finally, the resulting SiO_2 NPs were centrifuged and redispersed in 20 mL of ethanol.

Second, the SiO_2@PEI NPs were prepared via a PEI self-assembly process under sonication.[2] In brief, 0.25g PEI were dissolved in 100 mL of deionized water to prepare PEI solution. Then, 0.2 mL of prepared SiO_2 NPs (10 mg/mL) was dispersed in the PEI solution under sonication for 30 min, during which PEI gradually self-assembled on the silica cores. The resulting SiO_2@PEI NPs were separated by centrifugation at 5500 rpm for 6 min. The SiO_2@PEI NPs were washed for twice with deionized water and resuspended in 10 mL of deionized water for future us.

Third, the prepared SiO_2@PEI NPs were added into 100 mL carboxyl-functionalized CdSe/ZnS QD solution (1 nM) and sonicated for 30 min. The process was repeated two times to ensure the full absorption of QDs on the SiO_2@PEI surface. Finally, the resulting SiO_2@PEI-QDs were separated and dispersed in 10 mL ethanol.

S1.5 Preparation of immuno-SiO_2@PEI-QDs

Immuno-SiO_2@PEI-QDs were prepared by conjugating anti-*S. typhi* antibody onto the SiO_2@PEI-QDs surface via carbodiimide chemistry.[3] In brief, 0.5 mL of SiO_2@PEI-QDs in MES solution (100 mM, pH 5.5) was mixed with 50 μL of EDC (10 mM) and 10 μL of sulfo-NHS (100 mM) at 30 °C for 15 min. After purification, carboxyl-activated SiO_2@PEI-QDs were incubated with 15 μg of anti-*S. typhi*

antibody at 30 °C for 2 h. Then, 100 μL of 10% BSA was added to block unreacted carboxyl sites of SiO_2@PEI-QDs. Finally, the as-prepared immuno-SiO_2@PEI-QDs were separated by centrifugation (4000 rpm, 6 min) and washed twice with PBST buffer (10 mM, pH 7.4, 0.05% Tween 20). The final product was resuspended with 500 μL of preservation solution containing BBS (10 mM, pH 8.0), 1% BSA (w/v), 0.1% PVP (w/v), 10% sucrose (w/v), and 0.5% Tween-20 (v/v), and then was dispensed onto the glass fibre paper and dried to prepare the conjugate pad.

S1.6 Preparation of fluorescent LFA strips

The SiO_2@PEI-QD-based fluorescent LFA strip comprised an absorbent pad, an NC membrane with 15 mm pore size (CN95), a conjugate pad with SiO_2@PEI-QD nanotags, and a sample loading pad (Scheme 1b). 0.6 mg/mL of anti-*S. typhi* (Catalog #ab8274) and polyclonal goat anti-mouse IgG antibody diluted with 10 mM PBS were spotted onto the NC membrane as the test and control lines, respectively, with a dispense rate of 0.1 μL/mm via a spraying platform (Biodot xyz5050). The as-prepared NC membrane was dried in a constant temperature oven at 37 °C for 2 h. Subsequently, the sample pad, conjugate pad, NC membrane, and absorbent pad were assembled onto a plastic backing card. Finally, the prepared strips were fitted into plastic cassettes and stored with desiccants at room temperature until use.

S1.7 Sample testing on SiO_2@PEI-QDs based LFA strips

The assay was carried at room temperature. The sample pad of a test strip was dipped into an aliquot of the sample (70 μL) for 2 min and then placed on a horizontal surface. The result was checked within 10 min after sample application. The fluorescence signal on the test line was recorded using a fluorescent reader (365 nm excitation). For each test strip, the process of fluorescence reading was performed three times and the fluorescence data were collected and averaged before use.

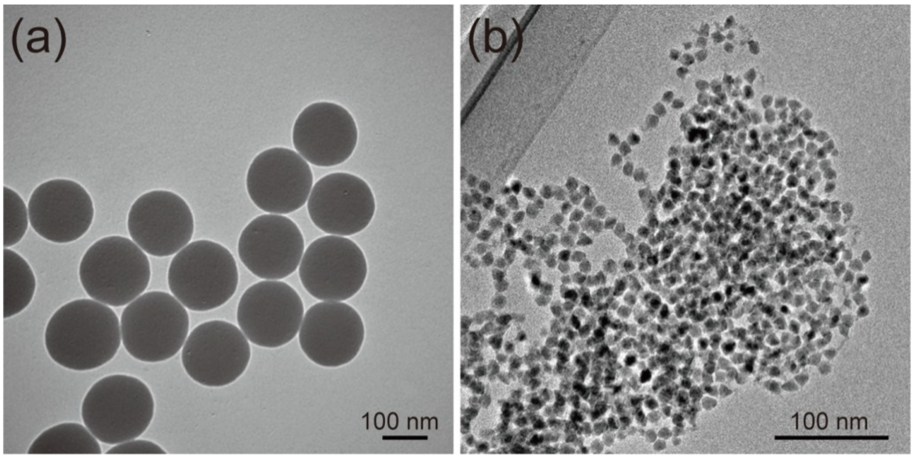

Fig. S1 TEM images of (a) the prepared SiO_2 NPs and (b) the commercial CdSe/ZnS-MPA QDs.

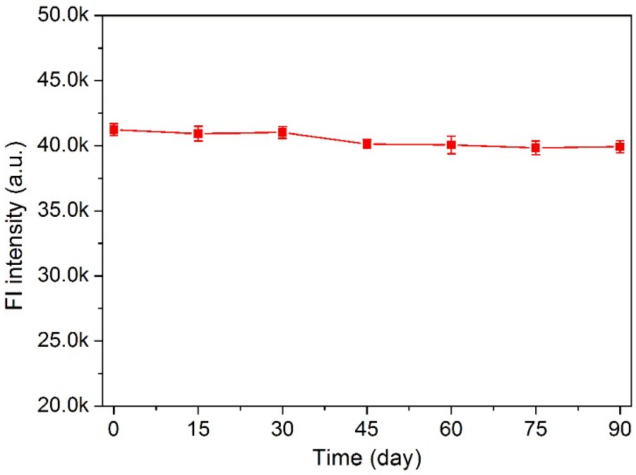

Fig. S2 Fluorescence intensity of SiO_2@PEI-QDs dispersed in ethanol against the storage time.

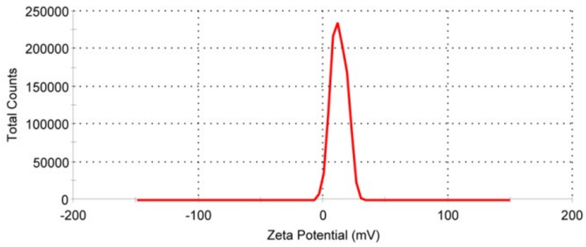

Fig. S3 Zeta potential of immuno-SiO_2@PEI-QDs in water.

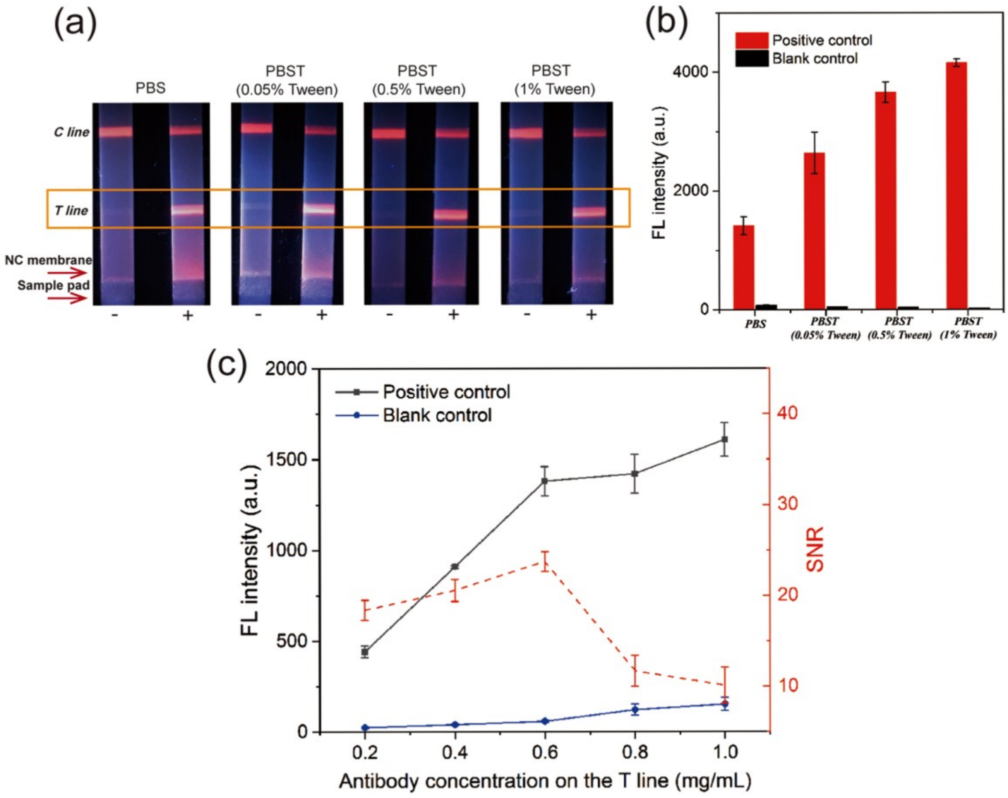

Fig. S4 (a-b) Optimization of running buffer solution and (c) antibody concentration on the T line for the SiO_2@PEI-QDs based fluorescent LFA strip. Error bars represent the standard deviation of three repetitive experiments.

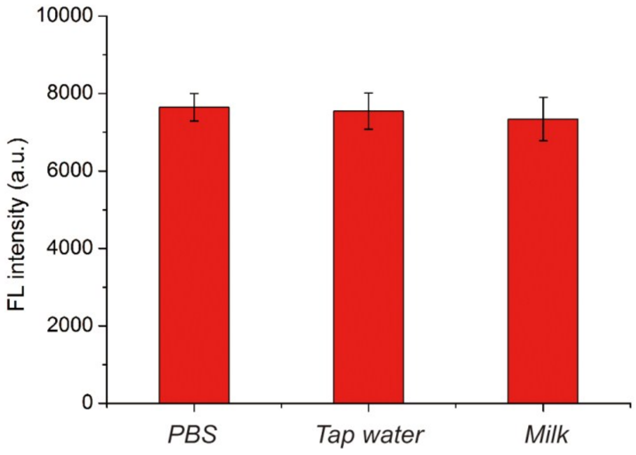

Fig. S5 Detection results of real samples spiked with *S. typhi* (10^6 cells/mL). Error bars are standard deviation of three repetitive experiments.

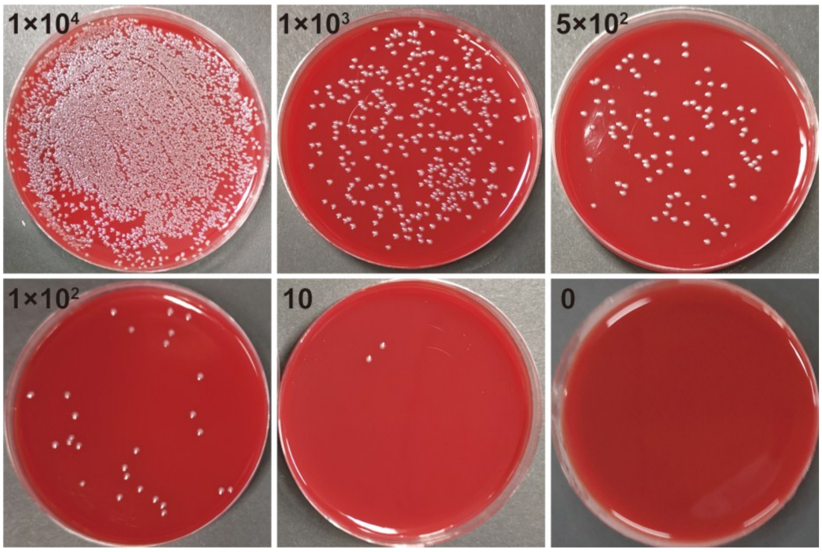

Fig. S6 The control experiments with plate counting method for *S. typhi* detection. 200 μL of the bacterial samples with different concentrations (10^4-0 cells/mL) was coated on the blood agar plates.

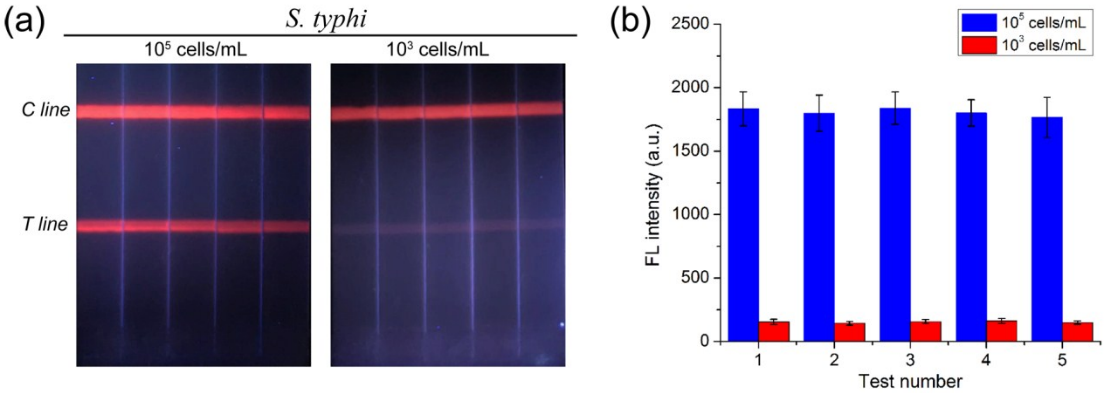

Fig. S7 Assay reproducibility of five tests of *S. typhi* at concentrations of 10^5 and 10^3 cells/mL. The error bars represent the standard deviations from three measurements.

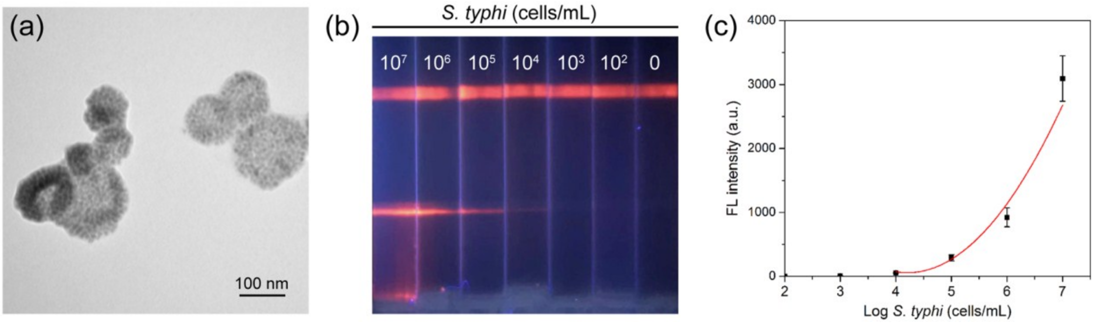

Fig. S8 Analytical performance of commercial QD microsphere-based fluorescent LFA strip. (a) TEM image of commercial QD microspheres. (b) fluorescence pictures

of QD microsphere-based LFA strip for *S. typhi* detection. (c) Calibration curve for *S. typhi* at a concentration range of 10^4-10^7 cells/mL.

References

1. T. Liu, D. Li, D. Yang and M. Jiang, *Colloid Surface A*, 2011, **387**, 17-22.
2. C. Wang, M. Li, Q. Li, K. Zhang, C. Wang, R. Xiao and S. Wang, *RSC Adv.*, 2017, **7**, 13138-13148.
3. K. Wang, Y. Wang, C. Wang, X. Jia, J. Li, R. Xiao and S. Wang, *RSC Adv.*, 2018, **8**, 30825-30831.
